# Antibodies to Human Herpesviruses and Rate of Incident Cardiovascular Events and All-Cause Mortality in the UK Biobank Infectious Disease Pilot Study

**DOI:** 10.1093/ofid/ofac294

**Published:** 2022-06-11

**Authors:** Petrina Chu, Sharon Louise Cadogan, Charlotte Warren-Gash

**Affiliations:** Department of Biostatistics & Health Informatics, Institute of Psychiatry, Psychology & Neuroscience, King’s College London, London, United Kingdom; Department of Non-Communicable Disease Epidemiology, London School of Hygiene & Tropical Medicine (LSHTM), London, United Kingdom; Department of Non-Communicable Disease Epidemiology, London School of Hygiene & Tropical Medicine (LSHTM), London, United Kingdom; Department of Non-Communicable Disease Epidemiology, London School of Hygiene & Tropical Medicine (LSHTM), London, United Kingdom

**Keywords:** human herpesvirus, mortality, myocardial infarction, stroke, UK Biobank

## Abstract

**Background:**

Associations between human herpesviruses (HHVs) and cardiovascular disease/mortality have been reported, but evidence is inconsistent. We investigated associations between 3 common herpesviruses and (1) incident stroke or myocardial infarction (MI) and (2) all-cause mortality.

**Methods:**

We included participants from the UK Biobank Infectious Disease pilot study with valid serum antibody (IgG) measurements taken at cohort entry (2006–2010) for herpes simplex virus type 1 (HSV1), varicella zoster virus (VZV), and cytomegalovirus (CMV). Linked hospital and mortality records up to December 30 2019 provided information on rates of (1) incident first stroke or MI and (2) all-cause mortality. Hazard ratios (HRs) from Cox proportional hazards regression models were used to assess relationships between (1) HHV seropositivity, (2) HHV titer and incident stroke/MI, and death outcomes. Fully adjusted models accounted for sociodemographic information (age, sex, ethnicity, education, deprivation quintile, birthplace, population density), baseline comorbidities (including diabetes and hypertension), smoking status, body mass index, and serum cholesterol.

**Results:**

Of 9429 study participants (56% female, 95% White, median age 58 years), 41% were seropositive for all 3 HHVs. Human herpesvirus seropositivity was not associated with stroke/MI (fully adjusted HRs and 95% confidence intervals [CIs]: HSV1 = 0.93 [CI, 0.72–1.22], VZV = 0.78 [CI, 0.51–1.20], CMV = 0.91 [CI, 0.71–1.16]) or all-cause mortality (HSV1 = 1.21 [CI, 1.00–1.47], VZV = 0.79 [CI, 0.58–1.07], CMV = 0.90 [CI, 0.76–1.06]). Human herpesvirus titers were not associated with outcomes.

**Conclusions:**

In this mostly White UK Biobank subset, neither HHV seropositivity nor titers were associated with stroke/MI or all-cause mortality.

## Teaser

In this secondary analysis of the UK Biobank Infectious Diseases pilot study, we found that 3 common human herpesviruses (herpes simplex virus type 1, herpes zoster, and cytomegalovirus) were not associated with rates of incident cardiovascular events or all-cause mortality.

Human herpesviruses (HHVs) are common deoxyribonucleic acid (DNA) viruses that can establish lifelong persistence in humans by evading or interfering with immune system detection and other signaling pathways [1, 2]. Globally, between 67% and 90% of people are estimated to be seropositive for common HHVs such as herpes simplex virus type 1 (HSV1), varicella zoster virus (VZV), or cytomegalovirus (CMV) [3–5]. In healthy individuals, infections are typically asymptomatic or subclinical but can cause serious complications such as encephalitis and meningitis in immunodeficient individuals where reactivation is more likely to occur [6, 7]. There has been growing interest in determining whether HHVs can increase the risk of acute cardiovascular events or all-cause mortality. Although the exact mechanisms are not well known, lifelong HHV infections could contribute to higher risks of cardiovascular events and mortality through chronic inflammatory pathways [8]. Because ischemic heart disease and stroke are the top 2 causes of death worldwide [9], investigating the effects of HHVs on cardiovascular health could help clinicians lower the global burden by identifying those at higher risk of developing cardiovascular disease.

Recent systematic reviews and longitudinal studies have produced mixed findings regarding the potential association between HHVs and cardiovascular disease or mortality. One review found that herpes zoster patients had twice the risk of stroke 4 weeks after zoster onset, but the risk of stroke returned to near-baseline levels after a year, whereas patients with recent CMV infection or reactivation (measured through immunoglobulin [Ig]M seropositivity) had over 5 times the risk of stroke compared to those without CMV IgM [10]. The association between past CMV infection (measured through IgG seropositivity) and stroke was less clear, and analysis of 5 prospective European cohorts found that CMV IgG seropositivity and high CMV IgG titer quartiles were not associated with increased risk of all-cause mortality or cardiovascular mortality [11]. Two other large cohort studies have reported conflicting evidence surrounding CMV seropositivity and mortality risks. In European Prospective Investigation into Cancer (EPIC)-Norfolk, higher CMV IgG titers were associated with increasing mortality rates even after adjusting for multiple confounders, whereas CMV seropositivity was associated with higher rates of all-cause mortality [12]. Researchers from the third US National Health and Nutrition Examination Survey (NHANES) reported an association between CMV exposure and all-cause mortality, but they found no evidence of an association between CMV seropositivity and mortality due to cardiovascular disease after adjusting for confounders [13]. Cytomegalovirus infection was associated with a higher risk of atherosclerosis in a recent meta-analysis [14], which could partly explain the observed relationship between CMV and cardiovascular events in some populations. Research into the cardiovascular effects of HSV1 and VZV (excluding clinical zoster) has been limited to small case-control studies and has not been investigated in detail. In addition, small sample sizes, study heterogeneity, and limited adjustment for confounding have continued to negatively impact current evidence surrounding CMV and cardiovascular or all-cause mortality.

This study aimed to investigate the association between 3 common herpesvirus exposures and (1) incident stroke or myocardial infarction and (2) all-cause mortality in a subset of the UK Biobank cohort, a large prospective study of older UK adults.

## METHODS

### Patient Consent Statement

All UK Biobank participants gave written informed consent, and the initial study was approved by the North West – Haydock Research Ethics Committee. The secondary data analysis was approved by the London School of Hygiene & Tropical Medicine ethics committee (Reference no. 21693).

### Study Population

UK Biobank is a prospective population-based cohort study that recruited over a half million adults aged 40–70 at baseline and living in the United Kingdom (UK). Baseline assessments were conducted from 2006 to 2010 across 22 assessment centers in England, Scotland, and Wales. Participants provided blood samples and physical measurements, responded to touchscreen questionnaires about sociodemographic and lifestyle factors, and underwent interviews with trained nurses. Participants consented to have hospital and death records linked to generate outcome data. In July 2016, 9724 participant baseline samples from the UK Biobank cohort were randomly selected to be assayed for various pathogens in the UK Biobank Infectious Diseases pilot study. Only participants from the Infectious Diseases pilot study with at least 1 valid measurement of herpesvirus antibodies were included in this analysis.

### Herpesvirus Antibody Measurements

The UK Biobank Infectious Diseases pilot study utilized a validated multiplex serology panel to assess IgG antibody levels for various pathogens, including HSV1, VZV, and CMV. Samples were processed using fluorescent bead-based high-throughput methods, antibody titer was expressed in median fluorescence intensity (MFI), and seropositivity cutoffs were chosen based on validated work [15]. Participants with MFI values equivocal to recommended cutoffs were considered seronegative. Antibody titers were grouped into tertiles among those seropositive, and tertile group was examined as a secondary exposure with seronegative patients as the baseline comparator. For CMV, tertiles were formed separately for each antigen among those seropositive.

### Ascertainment of Study Outcomes

The primary outcome was first incident stroke or myocardial infarction (MI) assessed using UK Biobank algorithms for linked death registration records, hospital records, and self-reports up to December 31, 2017 [16, 17]. After this date, the linked hospital records were used to identify hospitalizations for stroke or MI using the same *International Classification of Diseases, Tenth Revision* codes as the UK Biobank algorithms up to December 31, 2019. For incident stroke, first stroke of any type (ischemic, hemorrhagic, or nonspecific) was considered (see Supplementary Table 1). The secondary outcome of all-cause mortality was assessed using linked death registration records up to December 31, 2019 [18].

### Covariates

The overall Index of Multiple Deprivation (IMD) divided into quintiles was used to assess multiple deprivation and was calculated using the participant’s postcode at baseline and matched to the appropriate IMD based on the year of recruitment. Home area population density (urban/town and fringe/rural/isolated) was calculated by combining participant postcodes with 2001 Census data. Country of birth (UK/Elsewhere) was assessed during participant interviews. Information on participant ethnicity (White/Other), educational qualifications (postsecondary, secondary, less than secondary level), smoking status (never/previous/current), and presence of longstanding illness/disability/infirmity were collected via touchscreen questionnaire self-responses. Serum cholesterol (mmol/L) and body mass index ([BMI] kg/m^2^) were measured during baseline visit physical assessments. The following clinical comorbidities were identified through standardized medical coding in linked medical records and registries at any time up to the baseline visit (see Supplementary Table 2), or self-report at baseline assessments: asthma, chronic obstructive pulmonary disorder, atrial fibrillation, hypertension, diabetes, chronic liver disease, chronic kidney disease, unspecified cancer, chronic neurological disease, chronic autoimmune disease, and other chronic cardiovascular diseases.

### Statistical Analysis

Key demographic, clinical, and lifestyle characteristics were examined by HHV serostatus at baseline, along with levels of missing data. Kappa measures of agreement were calculated to assess the level of repeatability between baseline and follow-up measurements of HHV serostatus for participants with samples from 2 visits.

Person-years at risk in the primary incident stroke/MI analysis were calculated from baseline visit date until the first event of death, loss to follow-up, first incident stroke or MI, or last date of observation (December 31, 2019). Person-years at risk in the secondary all-cause mortality analysis were calculated from baseline visit date until the first event of death, loss to follow-up, or last date of observation (December 31, 2019). Cox proportional hazards regression models were used to calculate hazard ratios (HRs) and 95% confidence intervals (CIs) for (1) incident stroke/MI and (2) all-cause mortality. Formal tests of interaction with time (defined as time in follow-up) were conducted to confirm that there was no evidence against the proportional hazards assumption.

Separate models were calculated for each HHV with seronegative participants as the baseline comparator group, starting with unadjusted models. Minimally adjusted models included age (in years) and sex as covariates. In addition to age and sex, fully adjusted models for each HHV included baseline comorbidities, longstanding illness, baseline BMI and cholesterol, overall IMD quintile, ethnicity, birthplace, educational level, and population density. Directed acyclic graphs (Supplementary Figure 1) were generated using current literature to inform confounder adjustment. To facilitate comparisons for all 3 HHVs, the same set of covariates were chosen. Missing data, “don’t know”, or “prefer not to answer” responses were excluded from the complete case analysis. When the odds of being a complete case are not associated with the outcome after considering covariates, complete case analysis can yield unbiased results [19].

We assessed HHV antibody tertiles for differences in unadjusted survival functions using trend tests, and then we repeated the main models for these secondary exposures. Age (in years) and sex were assessed as potential effect modifiers for HHV seropositivity in exploratory analyses using interaction terms.

The first sensitivity analysis repeated the main models using the first measurement of HHV antibody levels to include the 260 individuals who did not have baseline HHV measurements available but had measurements from a repeat visit that were available for analysis. The second sensitivity analysis repeated the main models using HHV antibody levels as a continuous variable because there are no clear best practices on categorizing HHV antibody levels. All statistical analyses were performed in Stata (version 17.0).

## RESULTS

**Figure 1.**
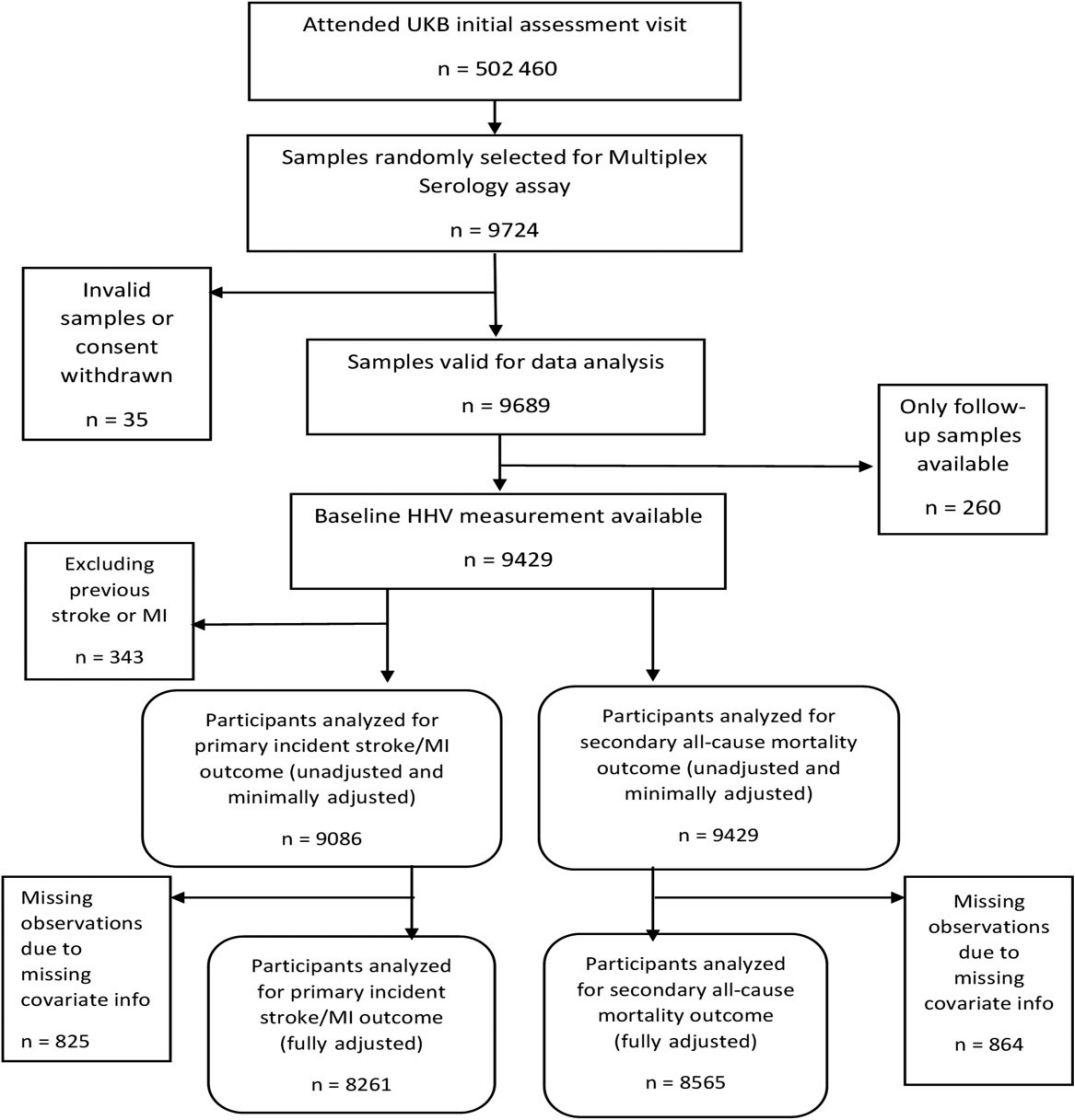
Flowchart depicting participant inclusion for study analysis. HHV, human herpesvirus; MI, myocardial infarction; UKB, UK Biobank.

Of 9429 participants with valid baseline HHV measurements (Figure 1), 6591 (70%) were seropositive for HSV1, 8714 (92%) were seropositive for VZV, and 5493 (58%) were seropositive for CMV. A total of 3906 participants (41%) were seropositive for all 3 HHVs. For participants with HHV measurements at baseline and follow-up visits, there were high levels of serostatus agreement (>90%) across all 3 HHVs.

For all HHVs, seropositive participants were more likely to be female and older than seronegative participants (Table 1). Participants seropositive for HSV1 or CMV were more likely to have higher average BMIs, have multiple baseline comorbidities, report current or previous smoking, and have lower educational levels compared with those seronegative for each virus. Overall, the study population was predominantly White (95%), UK-born (91%), and living in urban areas (85%).

**Table 1.** Baseline Characteristics of UK Biobank Infectious Disease Pilot Study Participants by Herpesvirus Serostatus^a^

Characteristic	Overall Cohort (n = 9689)	HSV1 Seronegative (n = 2838, 30%)	HSV1 Seropositive (n = 6591, 70%)	VZV Seronegative (n = 715, 7.6%)	VZV Seropositive (n = 8714, 92%)	CMV Seronegative (n = 3936, 42%)	CMV Seropositive (n = 5493, 58%)
Mean age, years (SD)	56.54 (8.14)	55.70 (8.14)	56.89 (8.14)	55.62 (8.23)	56.61 (8.15)	55.24 (8.29)	57.46 (7.93)
Sex							
Female	5419 (56)	1539 (54)	3737 (57)	462 (65)	4814 (55)	2142 (54)	3134 (57)
Male	4270 (44)	1299 (46)	2854 (43)	253 (35)	3900 (45)	1794 (46)	2359 (43)
Ethnicity							
White	9135 (94)	2736 (96)	6147 (93)	678 (95)	8205 (94)	3869 (98)	5014 (91)
Other	510 (5.3)	85 (3.0)	417 (6.3)	36 (5.0)	467 (5.4)	53 (1.4)	449 (8.2)
Missing (%)	44 (0.5)	17 (0.6)	27 (0.4)	1 (0.1)	43 (0.5)	14 (0.4)	30 (0.6)
IMD Quintile							
1st	1897 (20)	668 (24)	1184 (18)	144 (20)	1708 (20)	855 (22)	997 (18)
2nd	1887 (20)	651 (24)	1184 (18)	141 (20)	1694 (19)	803 (20)	1032 (19)
3rd	1889 (20)	583 (21)	1246 (19)	131 (18)	1698 (19)	776 (20)	1053 (19)
4th	1889 (20)	474 (17)	1366 (21)	143 (20)	1697 (19)	742 (19)	1098 (20)
5th	1885 (20)	385 (14)	1449 (23)	143 (20)	1692 (19)	656 (17)	1178 (21)
Missing (%)	242 (2.5)	77 (2.7)	162 (2.5)	13 (1.8)	226 (2.6)	104 (2.6)	135 (2.5)
Birthplace							
UK	8842 (91)	2646 (93)	5953 (91)	642 (90)	7957 (91)	3726 (95)	4873 (89)
Elsewhere	828 (8.5)	191 (6.7)	620 (9.4)	70 (9.8)	741 (8.5)	208 (5.3)	603 (11)
Missing (%)	19 (0.2)	1 (.04)	18 (0.3)	3 (0.4)	16 (0.2)	2 (0.1)	17 (0.3)
Educational Level							
Postsecondary	4262 (44)	1497 (53)	2619 (40)	308 (43)	3808 (44)	1824 (46)	2292 (42)
Secondary	3665 (38)	1079 (38)	2506 (39)	288 (40)	3297 (38)	1576 (40)	2009 (37)
Less than secondary	1647 (17)	250 (8.9)	1374 (21)	107 (15)	1517 (17)	506 (13)	1118 (20)
Missing (%)	115 (1.2)	12 (0.4)	92 (1.4)	12 (1.7)	92 (1.1)	30 (0.8)	74 (1.4)
Population Density							
Urban	8160 (84)	2302 (81)	5623 (85)	590 (83)	7335 (84)	3245 (82)	4680 (85)
Town and fringe	706 (7.3)	235 (8.3)	461 (7.0)	53 (7.4)	643 (7.4)	294 (7.5)	402 (7.3)
Rural	518 (5.4)	198 (7.0)	310 (4.7)	51 (7.1)	457 (5.2)	254 (6.5)	254 (4.6)
Isolated	207 (2.1)	72 (2.5)	130 (2.0)	15 (2.1)	187 (2.2)	98 (2.5)	104 (1.9)
Missing (%)	98 (1.0)	31 (1.1)	67 (1.0)	6 (0.8)	92 (1.1)	45 (1.1)	53 (1.0)
Smoking Status							
Never	5372 (56)	1703 (60)	3507 (53)	411 (58)	4799 (55)	2264 (58)	2946 (54)
Previous	3307 (34)	900 (32)	2318 (35)	241 (34)	2977 (34)	1264 (32)	1954 (36)
Current	956 (9.9)	228 (8.0)	721 (11)	61 (8.5)	888 (10.2)	396 (10.1)	553 (10.1)
Missing (%)	54 (0.6)	7 (0.3)	45 (0.7)	2 (0.3)	50 (0.6)	12 (0.3)	40 (0.7)
Clinical Comorbidities							
Asthma	1129 (12)	332 (12)	764 (12)	86 (12)	1010 (12)	446 (11)	650 (12)
COPD	177 (1.8)	43 (1.5)	133 (2.0)	15 (2.1)	161 (1.9)	59 (1.5)	117 (2.1)
Atrial fibrillation	150 (1.6)	40 (1.4)	108 (1.6)	10 (1.4)	138 (1.6)	66 (1.7)	82 (1.5)
Hypertension	922 (9.5)	194 (6.8)	708 (11)	60 (8.4)	842 (9.7)	322 (8.2)	580 (10.6)
Other CV disease	609 (6.3)	130 (4.6)	465 (7.1)	36 (5.0)	559 (6.4)	216 (5.5)	379 (6.9)
Diabetes	494 (5.1)	106 (3.7)	373 (5.7)	35 (4.9)	444 (5.1)	168 (4.3)	311 (5.7)
Liver disease	99 (1.0)	24 (0.9)	73 (1.1)	7 (1.0)	90 (1.0)	38 (1.0)	59 (1.1)
Renal disease	110 (1.1)	34 (1.2)	75 (1.1)	8 (1.1)	101 (1.2)	46 (1.2)	63 (1.2)
Cancer	891 (9.2)	236 (8.3)	634 (9.6)	71 (9.9)	799 (9.2)	355 (9.0)	515 (9.4)
Neurological disease	69 (0.7)	24 (0.9)	43 (0.7)	6 (0.8)	61 (0.7)	35 (0.9)	32 (0.6)
Clinical Comorbidities							
Autoimmune disease	250 (2.6)	66 (2.3)	178 (2.7)	21 (2.9)	223 (2.6)	96 (2.4)	148 (2.7)
Other longstanding illness^b^	3062 (32)	876 (31)	2108 (32)	208 (30)	2776 (32)	1203 (31)	1781 (33)
Clinical Biomarkers							
Mean BMI, kg/m^2^ (SD)	27.31 (4.79)	26.75 (4.71)	27.58 (4.82)	27.00 (4.87)	27.35 (4.80)	26.96 (4.75)	27.59 (4.83)
Missing (%)	32 (0.3)	7 (0.3)	25 (0.4)	2 (0.3)	30 (0.3)	11 (0.3)	21 (0.4)
Mean cholesterol, mmol/L (SD)	5.71 (1.16)	5.75 (1.16)	5.69 (1.17)	5.78 (1.20)	5.70 (1.16)	5.72 (1.16)	5.70 (1.17)
Missing (%)	154 (1.6)	36 (1.3)	83 (1.3)	12 (1.7)	107 (1.2)	52 (1.3)	67 (1.2)

Abbreviations: BMI, body mass index; CMV, cytomegalovirus; COPD, chronic obstructive pulmonary disorder; CV, cardiovascular; HSV1, herpes simplex virus type 1; IMD, index of multiple deprivation; SD, standard deviation; UK, United Kingdom; VZV, varicella zoster virus.

a Column percentages given for categorical values. For continuous variables, means reported along with SD.

b Missing or “don’t know”/“prefer not to answer” responses for 260 participants.

There were 9086 participants in the unadjusted primary analysis for incident stroke/MI after excluding participants with previous stroke/MI (Figure 1), and there were 9429 participants in the unadjusted secondary all-cause mortality analysis. Baseline age and sex were fully observed. Low levels of missing data on other covariates (ranging from 0.2% for birthplace to 2.5% for IMD) meant slightly fewer observations in fully adjusted models. The odds of being a complete case were not associated with outcomes after considering covariates. Of the 318 first incident strokes/MIs over a mean follow-up time of 10.6 years (standard deviation [SD] = 1.7 years), 21 were fatal events (same death date as stroke/MI date). There were 679 deaths due to all-causes over a mean follow-up time of 10.7 years (SD = 1.5 years).

**Figure 2.**
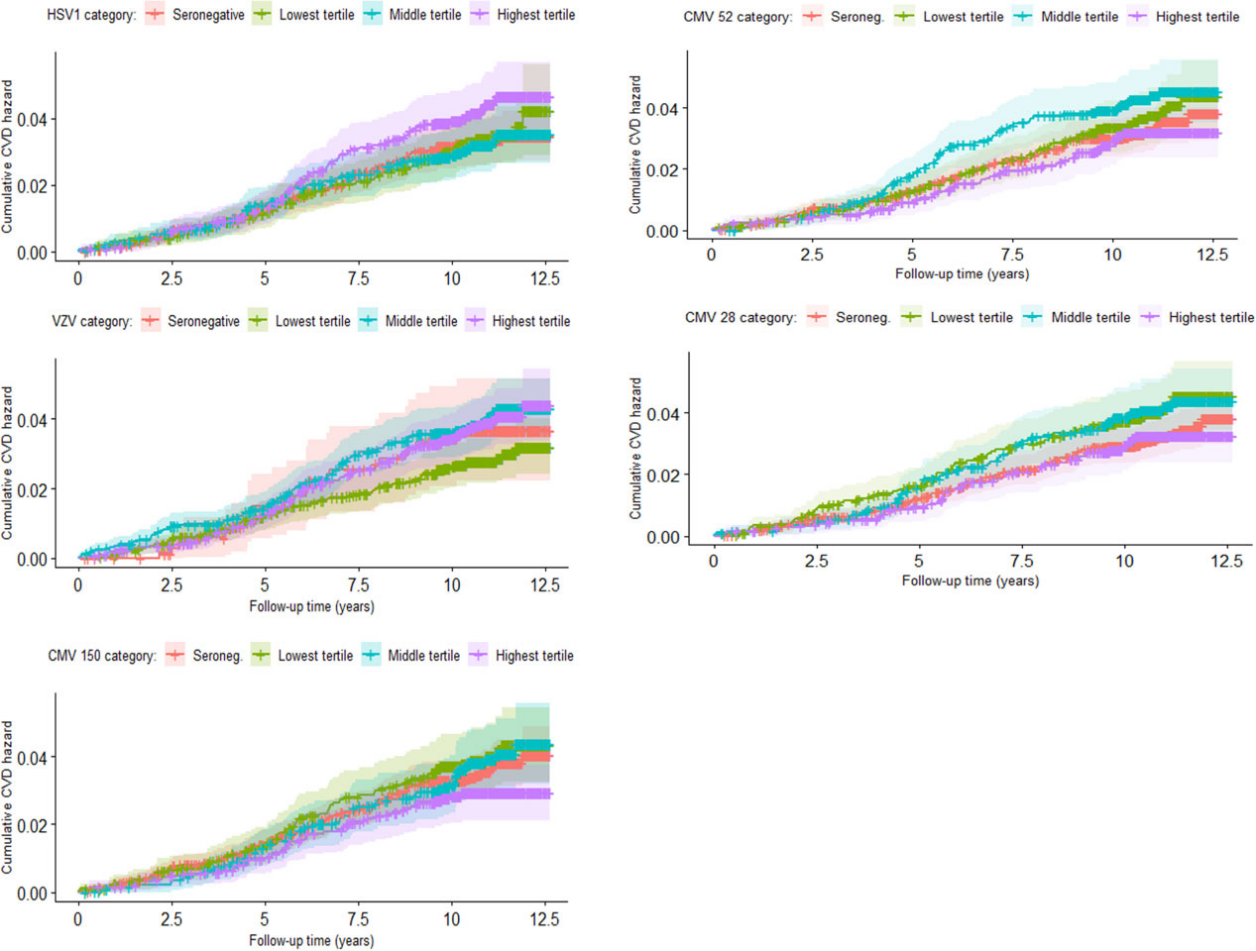
Unadjusted cumulative hazards by herpesvirus antigen for the first incident cardiovascular event ([CVD] stroke or myocardial infarction) event in UK Biobank Infectious Diseases Pilot study participants. CMV, cytomegalovirus; HSV1, herpes simplex virus type 1; VZV, varicella zoster virus.

Human herpesvirus seropositivity was not associated with stroke or MI in any model (Table 2). The fully adjusted HRs for stroke or MI were 0.93 (95% CI, 0.72‒1.22) for HSV1, 0.78 (95% CI, 0.51‒1.20) for VZV, and 0.91 (95% CI, 0.71‒1.16) for CMV. There was no clear pattern across increasing HHV antibody tertiles in unadjusted analyses (Figure 2), and adjusted analyses indicated that there was no association between higher tertiles and rates of stroke/MI (Supplementary Table 3). There was no evidence for interaction between serostatus and sex, but exploratory analyses indicated that there was some evidence of interaction between age at baseline and serostatus, even in the full models (likelihood-ratio test *P* values for interaction for the following: HSV1, *P* < .0001; VZV, *P* = .0042; and CMV, *P* = .002).

**Table 2.** Results of Cox Proportional Hazards Regression Models Investigating the Effects of Herpesvirus Seropositivity on Incident Cardiovascular Disease and All-Cause Mortality in UK Biobank Infectious Diseases Pilot Study

	Herpesvirus Status	Unadjusted Model, HR (95% CI)	Number of Observations	Minimally Adjusted Model^a^, HR (95% CI)	Number of Observations	Fully Adjusted Model^b^, HR (95% CI)	Number of Observations
Primary Outcome: Incident Stroke or Myocardial Infarction	HSV1 seropositive	1.12 (0.86–1.41)	9086	1.05 (0.82–1.34)	9086	0.93 (0.72–1.22)	8261
	VZV seropositive	0.98 (0.65–1.49)	9086	0.84 (0.55–1.28)	9086	0.78 (0.51–1.20)	8261
	CMV seropositive	1.04 (0.83–1.30)	9086	0.93 (0.74–1.17)	9086	0.91 (0.71–1.16)	8261
Secondary Outcome: All-Cause Mortality	Herpesvirus Status	Unadjusted Model, HR (95% CI)	Number of Observations	Minimally Adjusted Model^a^, HR (95% CI)	Number of Observations	Fully Adjusted Model^b^, HR (95% CI)	Number of Observations
	HSV1 seropositive	1.44 (1.20–1.72)	9429	1.31 (1.09–1.57)	9429	1.21 (0.997–1.47)	8565
	VZV seropositive	0.97 (0.73–1.28)	9429	0.82 (0.61–1.08)	9429	0.79 (0.58–1.07)	8565
	CMV seropositive	1.16 (0.99–1.36)	9429	0.99 (0.85–1.16)	9429	0.90 (0.76–1.06)	8565

Abbreviations: CI, confidence interval; CMV, cytomegalovirus; HR, hazard ratio; HSV1, herpes simplex virus type 1; VZV, varicella zoster virus.

a Adjusted for sex and age at baseline.

b Adjusted for sex, age, ethnicity, overall IMD quintile, birthplace, education, population density, smoking status, BMI, cholesterol, and clinical covariates and other longstanding illnesses at baseline.

**Figure 3.**
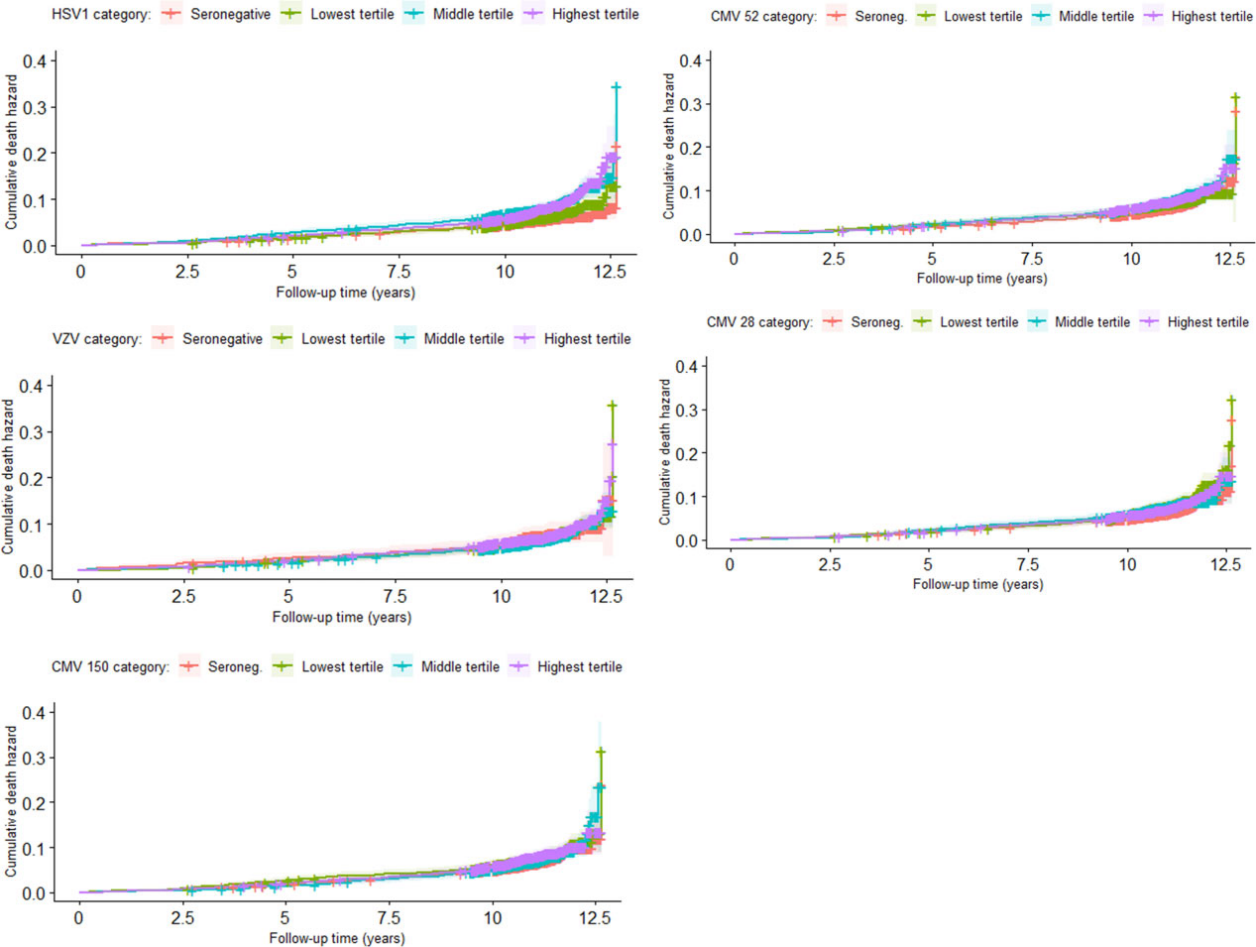
Unadjusted cumulative hazards by herpesvirus antigen for all-cause mortality in UK Biobank Infectious Diseases Pilot study participants. CMV, cytomegalovirus; HSV1, herpes simplex virus type 1; VZV, varicella zoster virus.

Human herpesvirus seropositivity was not associated with the secondary outcome of all-cause mortality in any model (Table 2). The fully adjusted HRs for all-cause mortality were 1.21 (95% CI, 1.00‒1.47) for HSV1, 0.79 (95% CI, 0.58‒1.07) for VZV, and 0.90 (95% CI, 0.76‒1.06) for CMV. In the unadjusted models for HHV tertiles, there was evidence that survival experiences across HSV1 tertiles was not the same (test for trend *P* < .0001), but the pattern was not apparent and there was no evidence that survival experiences differed across VZV or CMV tertiles (Figure 3). In adjusted analyses, there was no evidence of association between higher tertiles and rates of all-cause mortality (Supplementary Table 3). There was no evidence of interaction between seropositivity and age, or seropositivity and sex.

Sensitivity analyses supported the findings of the main analyses. When the first measurement of HHV antibodies taken either at baseline or at first follow up visit was used, no association was found between HHV seropositivity and incident stroke/MI (Supplementary Table 4). The fully adjusted HRs for stroke or MI were 0.96 (95% CI, 0.73–1.24) for HSV1, 0.77 (95% CI, 0.50–1.17) for VZV, and 0.94 (95% CI, 0.74–1.20) for CMV. When the first measurement of HHV antibodies was used in all-cause mortality analyses, there was weak evidence that HSV1 seropositivity was associated with higher rates of all-cause mortality (*P* = .0419). The fully adjusted HRs for all-cause mortality were 1.22 (95% CI, 1.00–1.48) for HSV1, 0.80 (95% CI, 0.59–1.08) for VZV, and 0.90 (95% CI, 0.76–1.07) for CMV. In sensitivity analyses treating HHV antibody levels continuously, there was no evidence of an association between higher HHV antibody levels and an increased rate of stroke/MI or all-cause mortality (Supplementary Table 5).

## DISCUSSION

In this representative sample of the UK Biobank cohort, we found no evidence of a significant association between HHV IgG seropositivity and higher first incident rates of (1) stroke/MI or (2) all-cause mortality. There was also no evidence that higher HHV IgG tertiles were associated with greater rates of either outcome.

Study findings agree with another recent analysis of CMV and cardiovascular disease in the UK Biobank cohort that found no association between CMV seropositivity and risk of ischemic heart disease or stroke after adjusting for confounders [20] and no evidence of a dose-response relationship across CMV tertiles. In a meta-analysis of 5 European cohorts, Chen et al [11] reported a pooled all-cause mortality HR of 1.05 (95% CI, 0.97–1.14) associated with CMV seropositivity and noted that no participants in the highest CMV IgG quartile reported greater risks of all-cause mortality compared to those seronegative.

In contrast, 2 large prospective cohort studies have reported significant associations between CMV seropositivity and mortality. The NHANES-III study of 14 153 Americans over 25 years old found that CMV seropositivity was associated with higher risk of all-cause mortality after adjusting for age, sex, ethnicity, country of origin, and clinical covariates (HR, 1.19; 95% CI, 1.01–1.41) but not cardiovascular-related mortality [13]. The EPIC-Norfolk study of 12 999 older UK adults aged 40–79 at baseline found that CMV seropositivity was associated with all-cause mortality after adjusting for age, sex, socioeconomic, and clinical confounders (HR, 1.15; 95% CI, 1.05–1.25) and that those in the highest CMV antibody tertile had the greatest risk of all-cause mortality (HR, 1.23; 95% CI, 1.09–1.37) compared to those seronegative [12]. Both studies had larger sample sizes than the UK Biobank Infectious Diseases pilot study, and there were more events observed in the NHANES-III and EPIC-Norfolk studies that may have generated greater study power to observe a difference in time to events. In addition, neither study adjusted for cancer at baseline, which could account for part of the observed association between CMV seropositivity and all-cause mortality. Cytomegalovirus has been linked to multiple cancer types (but is not believed to be oncogenic) and may play a role in the modulation of tumor malignancy [2, 21]. In smaller prospective studies conducted in older adults, differing results have been reported. A nested case-control study using 643 healthy male case-control pairs from the US Physicians’ Health Study found that neither CMV nor HSV (type unspecified) IgG seropositivity were associated with increased risks of atherothrombotic events [22], whereas analysis of 635 community-dwelling women in Maryland found that participants in the highest quartile of CMV IgG antibody levels were independently associated with having higher 5-year mortality rates [23]. Further research using large prospective cohort studies that establish HHV infection before cardiovascular events is needed to establish evidence for a dose-response relationship and/or evidence for effect modification by sex.

The systematic review by Forbes et al [10] found no evidence between past HSV1 or VZV infection and stroke. Although previous studies have reported a strong association between herpes zoster and stroke [24], this study utilized a serological definition where seropositivity occurs due to childhood chickenpox, whereas herpes zoster is a clinical illness caused by VZV reactivation, which may be more likely to result in inflammatory complications. There are no predefined cutoffs for identifying participants with zoster using the Multiplex Serology assay. In addition, a recent prospective study investigating VZV viral DNA and IgG antibody titers in participants with zoster found that VZV reactivation increased initial antibody levels that gradually fell in the months after reactivation, suggesting that antibody levels alone cannot reliably distinguish individuals with and without zoster [25]. Compared to CMV, there are fewer studies investigating the effects of higher HSV1 antibody titers on risk of myocardial infarction or other cardiovascular diseases. A recent meta-analysis of case-control studies found that HSV1 infection was associated with higher odds of atherosclerosis (odds ratio, 1.77; 95% CI, 1.40–2.23), although only unadjusted estimates from studies were used, and the authors noted a high degree of heterogeneity across HSV1 case-control studies [26]. When adjusting for age, sex, and multiple clinical covariates, HSV1 seropositivity was not associated with higher risks of myocardial infarction or death (relative hazard, 1.57; 95% CI, 0.88–2.80) in a small cohort of 890 patients undergoing coronary angiography [27].

In addition to a relatively large sample size and long follow-up period, this study had several strengths. Loss to follow-up was minimal, and data on exposures and outcomes were obtained using validated methods. The Multiplex Serology assays are highly specific, sensitive, and had high agreement index values with reference assays [15]. Human herpesvirus IgG antibody levels are typically stable indicators of previous exposure [22, 28], and tests of measurement repeatability for participants with baseline and follow-up visit HHV measurements indicated high levels of agreement. European electronic health records (EHRs) generally have high sensitivity and positive predictive values for stroke and MI [29]. Although serological assays often cannot distinguish between antibodies generated in response to vaccines and antibodies generated in response to natural infection, this is only a negligible issue in this study because the herpes zoster vaccine for older adults was not introduced in the UK until 2013, and there are no routine vaccines for HSV1 or CMV. The UK Biobank dataset contains robust information on multiple confounders, but residual confounding is possible if there are undiagnosed comorbidities not captured by electronic health records.

This study had several limitations. Although HHV IgG levels may approximate the frequency of infection reactivations [13, 25, 30] and could be correlated with HHV-specific immune cell counts [31, 32], the absence of follow-up measurements for the cohort majority may result in the loss of information regarding changes in tertile groups or reactivation frequency over time. If participants experienced more frequent reactivation episodes in later years of follow-up, a stronger association between HHV antibody levels and (1) incident stroke or MI and (2) all-cause mortality could have been missed. In addition, higher HHV antibody levels could be the result of underlying stress or immune dysfunction rather than actual infection burden [33]. We lacked information on other causes of immunosuppression, which might impact serological responses to HHV infections. However, the estimated prevalence of severe immunosuppression (defined as the presence of organ transplantation, chemoradiotherapy, cell-mediated immunosuppressive conditions, bone marrow transplantation, and long-term oral steroid use by codes in linked EHRs and self-report in the 5 years before baseline) in UK Biobank participants was only 3.2% [34], so this is unlikely to be a major source of bias.

In addition, study results may not be generalizable and limited to the majority White, UK-born population. Consistent with other reports of higher CMV seroprevalences in non-White populations [35], 88% of non-White study participants were CMV seropositive compared to 55% of White study participants (Supplementary Table 6). UK Biobank cohort participants may be healthier and wealthier than the general population [36]. In addition, there are concerns that any selection bias present due to nonrandom voluntary participation in UK Biobank could lead to collider bias that affect interpretability of HRs [37]. Although this may affect generalizability and interpretation of results, some research has shown that exposure-outcome relationships identified in UK Biobank are similar to those found in representative datasets such as the Health Survey for England [33]. Nevertheless, as non-White populations in lower- and middle-income countries adopt Westernized diets and lifestyles, it will become increasingly important to conduct large representative studies investigating potential risk factors for cardiovascular disease in other settings. Whether herpesvirus exposures will appear as risk factors for cardiovascular disease or mortality in other populations remains unclear.

## CONCLUSIONS

In summary, there was no evidence of an association between higher herpesvirus antibody levels and increased risk of (1) stroke or MI or (2) all-cause mortality in this sample of healthy majority-White UK adults. Repeat assessments of IgG antibody or viral DNA levels over time could better capture herpesvirus exposure, along with studies of pathogen burden that combine several chronic virus exposures. Finally, similar prospective cohort studies in other settings are needed to assess the long-term effects of herpesvirus infections in different populations.

## Supplementary Data

Supplementary materials are available at *Open Forum Infectious Diseases* online. Consisting of data provided by the authors to benefit the reader, the posted materials are not copyedited and are the sole responsibility of the authors, so questions or comments should be addressed to the corresponding author.

## Supplementary Material

ofac294_Supplementary_Data
