# Supplementary material for: Antibodies to Human Herpesviruses and Rate of Incident Cardiovascular Events and All-Cause Mortality in the UK Biobank Infectious Disease Pilot Study
Source: Open Forum Infect Dis. 2022 Jun 11;9(7):ofac294. doi: 10.1093/ofid/ofac294 (PMC9301583; doi:10.1093/ofid/ofac294)
Supplement: ofac294_Supplementary_Data [file ofac294_supplementary_data.zip › supplementary_table1.docx]

| **Supplementary Table 1: List of UK Biobank codes for stroke and myocardial infarction.** |
| --- |
| Stroke - ICD 9 |
| 430.X (SH), 431.X (IH), 434.X (occlusion of cerebral arteries), 434.0 (cerebral thrombosis), 434.1 (cerebral embolism), 434.9 (cerebral artery occlusion, unspecified), 436.X (acute ill-defined cerebrovascular disease) |
| Stroke - ICD 10 |
| I60 and I60.0 ‒ I60.9 (SH), I61 and I61.0 ‒ I61.6/I61.8/I61.9 (IH), I63 and I63.0 ‒ I63.6/I63.8/I63.9 (cerebral infarction), I64.X (stroke, not specified as haemorrhage or infarction) |
| MI - ICD 9 |
| 410 and 410.0 ‒ 410.9 (acute MI), 411 (other acute ischemic heart disease), 411.0 (post MI syndrome), 411.1 (intermediate coronary syndrome), 411.8 (Other), 412.X (old MI), 429.79 (ill-defined descriptions and complications of heart disease - other) |
| MI - ICD 10 |
| I21 and I21.0 ‒ I21.4/I21.9 (acute MI), I22 and I22.0/I22.1/I22.8/I22.9 (subsequent MI), I23 and I23.0 ‒ I23.6/I23.8 (current complications following acute MI), I24.1 (Dressler syndrome), I25.2 (old MI) |
| Stroke defined as stroke of any type, includes ischemic stroke (IS), intracerebral haemorrhage (IH), and subarachnoid haemorrhage (SH). MI includes ST elevation MI (STEMI) and non-ST elevation MI (NSTEMI). Abbreviations: MI, myocardial infarction; ICD, International Classification of Diseases. |
