# Supplementary material for: Antibodies to Human Herpesviruses and Rate of Incident Cardiovascular Events and All-Cause Mortality in the UK Biobank Infectious Disease Pilot Study
Source: Open Forum Infect Dis. 2022 Jun 11;9(7):ofac294. doi: 10.1093/ofid/ofac294 (PMC9301583; doi:10.1093/ofid/ofac294)
Supplement: ofac294_Supplementary_Data [file ofac294_supplementary_data.zip › supplementary_table2.docx]

**Supplementary Table 2: Clinical covariate descriptions and identification codes**

| **Covariate** | **Description (ICD 9 code)** | **Description (ICD 10 code)** |
| --- | --- | --- |
| Asthma | Asthma (493), extrinsic asthma (493.0), intrinsic asthma (493.1), chronic obstructive asthma (493.2), other forms of asthma (493.8), unspecified asthma (493.9) | Asthma (J45), predominantly allergic asthma (J45.0), nonallergic asthma (J45.1), mixed asthma (J45.8), unspecified asthma (J45.9), status asthmaticus (J46.X) |
| COPD | Emphysema (492), emphysema bleb (492.0), other emphysema (492.8), chronic airway obstruction (496.X) | Emphysema (J43), MacLeod syndrome (J43.0), panlobular emphysema (J43.1), centrilobular emphysema (J43.2), other emphysema (J43.8), unspecified emphysema (J43.9), other COPD (J44), COPD with acute lower respiratory infection (J44.0), COPD with unspecified acute exacerbation (J44.1), other specified COPD (J44.8), unspecified COPD (J44.9) |
| Atrial fibrillation and flutter | … | Atrial fibrillation and flutter (I48) |
| Hypertension | … | Essential hypertension (I10), hypertensive heart disease (I11), hypertensive renal disease (I12), hypertensive heart and renal disease (I13), secondary hypertension (I14) |
| Diabetes | … | Insulin-dependent diabetes mellitus (E10), non-insulin-dependent diabetes mellitus (E11), malnutrition-related diabetes mellitus (E12), other specified diabetes mellitus (E13), insulin-dependent diabetes mellitus (E14) |
| Chronic liver disease | … | Alcoholic liver disease (K70), toxic liver disease (K71), fibrosis and cirrhosis of liver (K74), other inflammatory liver diseases (K75), other disease of liver (K76), liver disorders in diseases classified elsewhere (K77) |
| Chronic kidney disease | … | Chronic renal failure (N18), end-stage renal disease reports as identified in UK Biobank algorithms |
| Other cardiovascular disease | … | Angina pectoris (I20), other acute ischemic heart diseases (I24), chronic ischemic heart disease (I25), heart failure (I50), atherosclerosis (I70), aortic aneurysm and dissection (I71), other aneurysm and dissection (I72), other peripheral vascular diseases (I743), arterial embolism and thrombosis (I74) |
| Chronic neurological disease | All cause dementia, motor neurone disease, all cause parkinsonism, and multiple sclerosis reports as identified in UK Biobank algorithms | All cause dementia, motor neurone disease, all cause parkinsonism, and multiple sclerosis reports as identified in UK Biobank algorithms |
| Autoimmune disorders | … | Seropositive rheumatoid arthritis (M05), other rheumatoid arthritis (M06), psoriatic and enteropathic arthropathies (M07), Crohn’s disease (K50), ulcerative colitis (K51), systemic lupus erythematosus (M32) |

Abbreviations: ICD, International Classification of Disease; COPD, chronic obstructive pulmonary disorder.
