# Supplementary material for: Antibodies to Human Herpesviruses and Rate of Incident Cardiovascular Events and All-Cause Mortality in the UK Biobank Infectious Disease Pilot Study
Source: Open Forum Infect Dis. 2022 Jun 11;9(7):ofac294. doi: 10.1093/ofid/ofac294 (PMC9301583; doi:10.1093/ofid/ofac294)
Supplement: ofac294_Supplementary_Data [file ofac294_supplementary_data.zip › supplementary_table3_tertiles.docx]

Table 3. Results of Cox proportional hazards regression models investigating the effects of herpesvirus antibody tertiles on incident cardiovascular disease and all-cause mortality in UK Biobank Infectious Diseases pilot study.

| **Primary outcome: incident cardiovascular disease (stroke or myocardial infarction)** | | | | |
| --- | --- | --- | --- | --- |
| Herpesvirus | Tertile | Crude model, HR (95% CI) | Minimally adjusted model^a^, HR (95% CI) | Fully adjusted model^b^, HR (95% CI) |
| HSV1 | Lowest tertile | 1.03 (0.75 ‒ 1.41) | 1.03 (0.75 – 1.41) | 0.96 (0.69 – 1.34) |
|  | Middle tertile | 0.98 (0.71 – 1.35) | 0.95 (0.69 – 1.30) | 0.76 (0.53 – 1.08) |
|  | Highest tertile | 1.30 (0.97 – 1.75) | 1.14 (0.85 – 1.54) | 1.07 (0.78 – 1.46) |
| VZV | Lowest tertile | 0.78 (0.50 – 1.24) | 0.73 (0.46 – 1.16) | 0.70 (0.44 – 1.12) |
|  | Middle tertile | 1.10 (0.71 – 1.71) | 0.95 (0.61 – 1.48) | 0.82 (0.52 – 1.30) |
|  | Highest tertile | 1.06 (0.68 – 1.64) | 0.85 (0.54 – 1.32) | 0.82 (0.52 – 1.29) |
| CMV pp150 | Lowest tertile | 1.11 (0.83 – 1.49) | 0.96 (0.71 – 1.29) | 0.92 (0.67 – 1.27) |
|  | Middle tertile | 1.06 (0.79 – 1.43) | 0.98 (0.72 – 1.32) | 0.97 (0.71 – 1.33) |
|  | Highest tertile | 0.81 (0.58 – 1.11) | 0.75 (0.54 – 1.04) | 0.64 (0.45 – 0.93) |
| CMV pp52 | Lowest tertile | 1.13 (0.83 – 1.53) | 1.02 (0.75 – 1.38) | 1.02 (0.74 – 1.41) |
|  | Middle tertile | 1.30 (0.97 – 1.74) | 1.13 (0.85 – 1.52) | 1.12 (0.82 – 1.54) |
|  | Highest tertile | 0.92 (0.67 – 1.27) | 0.85 (0.61 – 1.17) | 0.81 (0.58 – 1.15) |
| CMV pp28 | Lowest tertile | 1.28 (0.95 – 1.72) | 1.10 (0.81 – 1.47) | 1.09 (0.80 – 1.49) |
|  | Middle tertile | 1.27 (0.95 – 1.71) | 1.14 (0.85 – 1.54) | 1.12 (0.81 – 1.54) |
|  | Highest tertile | 0.97 (0.70 – 1.33) | 0.91 (0.66 – 1.25) | 0.82 (0.58 – 1.17) |
| **Secondary outcome: all-cause mortality** | | | | |
| Herpesvirus | Tertile | Crude model, HR (95% CI) | Minimally adjusted model^a^, HR (95% CI) | Fully adjusted model^b^, HR (95% CI) |
| HSV1 | Lowest tertile | 1.14 (0.91 – 1.44) | 1.13 (0.90 – 1.43) | 1.16 (0.91 – 1.48) |
|  | Middle tertile | 1.61 (1.30 – 1.99) | 1.49 (1.21 – 1.84) | 1.32 (1.04 – 1.66) |
|  | Highest tertile | 1.55 (1.25 – 1.92) | 1.29 (1.05 – 1.60) | 1.17 (0.93 – 1.47) |
| VZV | Lowest tertile | 0.96 (0.71 – 1.31) | 0.89 (0.65 – 1.21) | 0.86 (0.62 – 1.19) |
|  | Middle tertile | 0.91 (0.67 – 1.24) | 0.77 (0.57 – 1.05) | 0.72 (0.52 – 1.00) |
|  | Highest tertile | 1.03 (0.76 – 1.39) | 0.79 (0.59 – 1.08) | 0.80 (0.58 – 1.10) |
| CMV pp150 | Lowest tertile | 1.15 (0.94 – 1.41) | 0.95 (0.77 – 1.16) | 0.93 (0.75 – 1.15) |
|  | Middle tertile | 1.08 (0.87 – 1.33) | 0.95 (0.77 – 1.17) | 0.84 (0.67 – 1.06) |
|  | Highest tertile | 1.15 (0.94 – 1.41) | 1.02 (0.83 – 1.25) | 0.91 (0.73 – 1.14) |
| CMV pp52 | Lowest tertile | 1.10 (0.89 – 1.37) | 0.99 (0.80 – 1.22) | 0.92 (0.74 – 1.16) |
|  | Middle tertile | 1.25 (1.02 – 1.53) | 1.04 (0.84 – 1.28) | 0.94 (0.75 – 1.17) |
|  | Highest tertile | 1.22 (1.00 – 1.50) | 1.06 (0.86 – 1.30) | 0.96 (0.77 – 1.19) |
| CMV pp28 | Lowest tertile | 1.28 (1.04 – 1.57) | 1.05 (0.86 – 1.29) | 0.95 (0.77 – 1.19) |
|  | Middle tertile | 1.18 (0.96 – 1.46) | 0.99 (0.80 – 1.22) | 0.91 (0.72 – 1.13) |
|  | Highest tertile | 1.17 (0.95 – 1.44) | 1.07 (0.87 – 1.31) | 0.94 (0.75 – 1.18) |

There were 9086 observations in the crude analyses for incident stroke/myocardial infarction, and 9429 observations in the crude analyses for all-cause mortality. Abbreviations: HSV1, herpes simplex virus type 1; VZV, varicella zoster virus; CMV, cytomegalovirus; HR, hazard ratio; CI, confidence interval.

^a^ Adjusted for sex and age at baseline. Number of observations = 9086 in stroke/myocardial infarction analysis, 9429 in all-cause mortality analysis.

^b^ Adjusted for sex, age, ethnicity, overall IMD quintile, birthplace, education, population density, smoking status, BMI, cholesterol, and clinical covariates and other longstanding illnesses at baseline. Number of observations = 8261 in stroke/myocardial infarction analysis, 8565 in all-cause mortality analysis.
