# Supplementary material for: Antibodies to Human Herpesviruses and Rate of Incident Cardiovascular Events and All-Cause Mortality in the UK Biobank Infectious Disease Pilot Study
Source: Open Forum Infect Dis. 2022 Jun 11;9(7):ofac294. doi: 10.1093/ofid/ofac294 (PMC9301583; doi:10.1093/ofid/ofac294)
Supplement: ofac294_Supplementary_Data [file ofac294_supplementary_data.zip › supplementary_table4_sens_firstmmt.docx]

Table 4. Results of sensitivity analyses using first herpesvirus antibody measurements in Cox proportional hazards regression models investigating the effects of herpesvirus seropositivity on incident cardiovascular disease and all-cause mortality in UK Biobank Infectious Diseases pilot study.

| **Primary outcome: incident cardiovascular disease (stroke or myocardial infarction)** | | | | | | |
| --- | --- | --- | --- | --- | --- | --- |
| Herpesvirus status | Unadjusted model, HR (95% CI) | Number of observations | Minimally adjusted model^a^, HR (95% CI) | Number of observations | Fully adjusted model^b^, HR (95% CI) | Number of observations |
| HSV1 seropositive | 1.14 (0.89 – 1.45) | 9335 | 1.08 (0.84 – 1.38) | 9335 | 0.96 (0.74 – 1.24) | 8463 |
| VZV seropositive | 0.96 (0.64 – 1.45) | 9335 | 0.83 (0.55 – 1.25) | 9335 | 0.77 (0.50 – 1.17) | 8463 |
| CMV seropositive | 1.07 (0.86 – 1.34) | 9335 | 0.96 (0.77 – 1.21) | 9335 | 0.94 (0.74 – 1.20) | 8463 |
| **Secondary outcome: all-cause mortality** | | | | | | |
| Herpesvirus status | Unadjusted model, HR (95% CI) | Number of observations | Minimally adjusted model^a^, HR (95% CI) | Number of observations | Fully adjusted model^b^, HR (95% CI) | Number of observations |
| HSV1 seropositive | 1.45 (1.21 – 1.73) | 9689 | 1.31 (1.10 – 1.57) | 9689 | 1.22 (1.00 – 1.48) | 8778 |
| VZV seropositive | 0.97 (0.73 – 1.29) | 9689 | 0.83 (0.63 – 1.10) | 9689 | 0.80 (0.59 – 1.08) | 8778 |
| CMV seropositive | 1.16 (1.00 – 1.36) | 9689 | 1.00 (0.85 – 1.16) | 9689 | 0.90 (0.76 – 1.07) | 8778 |

Abbreviations: HSV1, herpes simplex virus type 1; VZV, varicella zoster virus; CMV, cytomegalovirus; HR, hazard ratio; CI, confidence interval.

^a^ Adjusted for sex and age at baseline.

^b^ Adjusted for sex, age, ethnicity, overall IMD quintile, birthplace, education, population density, smoking status, BMI, cholesterol, and clinical covariates and other longstanding illnesses at baseline.
