# Supplementary material for: Antibodies to Human Herpesviruses and Rate of Incident Cardiovascular Events and All-Cause Mortality in the UK Biobank Infectious Disease Pilot Study
Source: Open Forum Infect Dis. 2022 Jun 11;9(7):ofac294. doi: 10.1093/ofid/ofac294 (PMC9301583; doi:10.1093/ofid/ofac294)
Supplement: ofac294_Supplementary_Data [file ofac294_supplementary_data.zip › supplementary_table5_sens_continuous.docx]

Table 5. Results of sensitivity analyses treating baseline herpesvirus antibody levels as continuous exposures in Cox proportional hazards regression models investigating the effects of herpesviruses on incident cardiovascular disease and all-cause mortality in UK Biobank Infectious Diseases pilot study.

| **Primary outcome: incident cardiovascular disease (stroke or myocardial infarction)** | | | | | | |
| --- | --- | --- | --- | --- | --- | --- |
| Herpesvirus antibody | Unadjusted model, beta (SE) | Number of observations | Minimally adjusted model^a^, beta (SE) | Number of observations | Fully adjusted model^b^, beta (SE) | Number of observations |
| HSV1 | 2.80 x 10^-5^ (1.85 x 10^-5^) | 9086 | 1.03 x 10^-5^ (1.84 x 10^-5^) | 9086 | -8.01 x 10^-8^ (2.01 x 10^-5^) | 8261 |
| VZV | 4.02 x 10^-5^ (4.47 x 10^-5^) | 9086 | -4.27 x 10^-6^ (4.64 x 10^-5^) | 9086 | -1.66 x 10^-5^ (4.93 x 10^-5^) | 8261 |
| CMV pp150 | -1.97 x 10^-5^ (2.89 x 10^-5^) | 9086 | -2.93 x 10^-5^ (2.94 x 10^-5^) | 9086 | -4.83 x 10^-5^ (3.21 x 10^-5^) | 8261 |
| CMV pp52 | -5.58 x 10^-6^ (1.67 x 10^-5^) | 9086 | -1.5 x 10^-5^ (1.71 x 10^-5^) | 9086 | -2.26 x 10^-5^ (1.87 x 10^-5^) | 8261 |
| CMV pp28 | -2.78 x 10^-5^ (3.53 x 10^-5^) | 9086 | -3.53 x 10^-5^ (3.63 x 10^-5^) | 9086 | -5.41 x 10^-5^ (3.97 x 10^-5^) | 8261 |
| **Secondary outcome: all-cause mortality** | | | | | | |
| Herpesvirus antibody | Unadjusted model, beta (SE) | Number of observations | Minimally adjusted model^a^, beta (SE) | Number of observations | Fully adjusted model^b^, beta (SE) | Number of observations |
| HSV1 | 5.68 x 10^-5^ (1.25 x 10^-5^) | 9429 | 3.23 x 10^-5^ (1.25 x 10^-5^) | 9429 | 1.25 x 10^-5^ (1.36 x 10^-5^) | 8565 |
| VZV | 5.10 x 10^-5^ (3.01 x 10^-5^) | 9429 | 2.08 x 10^-6^ (3.16 x 10^-5^) | 9429 | 4.43 x 10^-5^ (3.26 x 10^-5^) | 8565 |
| CMV pp150 | 2.46 x 10^-5^ (1.86 x 10^-5^) | 9429 | 7.24 x 10^-6^ (1.89 x 10^-5^) | 9429 | -1.90 x 10^-5^ (2.05 x 10^-5^) | 8565 |
| CMV pp52 | 2.22 x 10^-5^ (1.11 x 10^-5^) | 9429 | 7.202 x 10^-6^ (1.14 x 10^-5^) | 9429 | -2.05 x 10^-6^ (1.24 x 10^-5^) | 8565 |
| CMV pp28 | 2.89 x 10^-5^ (2.25 x 10^-5^) | 9429 | 1.89 x 10^-5^ (2.34 x 10^-5^) | 9429 | -6.14 x 10^-6^ (2.55 x 10^-5^) | 8565 |

Abbreviations: HSV1, herpes simplex virus type 1; VZV, varicella zoster virus; CMV, cytomegalovirus; SE, standard error.

^a^ Adjusted for sex and age at baseline.

^b^ Adjusted for sex, age, ethnicity, overall IMD quintile, birthplace, education, population density, smoking status, BMI, cholesterol, and clinical covariates and other longstanding illnesses at baseline.
