# Supplementary material for: Antibodies to Human Herpesviruses and Rate of Incident Cardiovascular Events and All-Cause Mortality in the UK Biobank Infectious Disease Pilot Study
Source: Open Forum Infect Dis. 2022 Jun 11;9(7):ofac294. doi: 10.1093/ofid/ofac294 (PMC9301583; doi:10.1093/ofid/ofac294)
Supplement: ofac294_Supplementary_Data [file ofac294_supplementary_data.zip › supplementary_table6_ethnic_HHVseroprev.docx]

Supplementary Table 6: Herpesvirus antibody seroprevalences across ethnic categories (White/Other) at baseline in UK Biobank Infectious Diseases pilot study.

| Serostatus | Overall (N=9429)^a^ | White (N= 9135)^b^ | Other (N=510) |
| --- | --- | --- | --- |
| HSV1 seropositive (n, % of N) | 6591 (70%) | 6147 (67%) | 417 (82%) |
| VZV seropositive (n, % of N) | 8714 (92%) | 8205 (90%) | 466 (91%) |
| CMV seropositive (n, % of N) | 5493 (58%) | 5014 (55%) | 449 (88%) |

Column percentages are given here. The category of ‘Other’ includes: Mixed/Other, Asian, Black, Chinese.

Abbreviations: HSV1, herpes simplex virus type 1; VZV, varicella zoster virus; CMV, cytomegalovirus.

^a^ There were 9429 participants (out of a total 9689 participants) with herpesvirus antibody measurements at baseline.

^b^ Information on ethnicity was available for 9645 participants.
