# Supplementary figures and images for: Antibodies to Human Herpesviruses and Rate of Incident Cardiovascular Events and All-Cause Mortality in the UK Biobank Infectious Disease Pilot Study
Source: Open Forum Infect Dis. 2022 Jun 11;9(7):ofac294. doi: 10.1093/ofid/ofac294 (PMC9301583; doi:10.1093/ofid/ofac294)

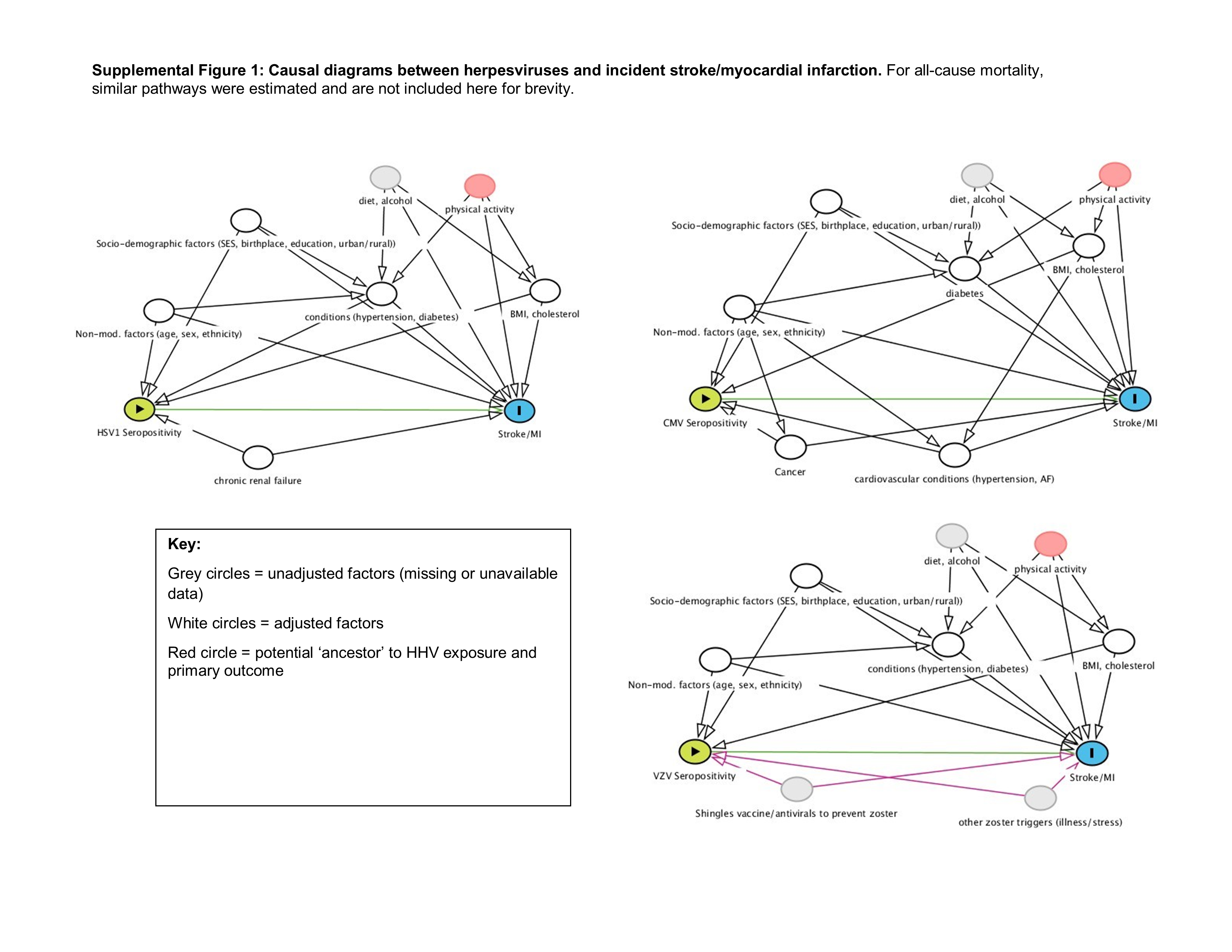

Supplement: ofac294_Supplementary_Data [file ofac294_supplementary_data.zip › supp_fig1_dag.tif]
